# Supplementary material for: Autophagy-Dependent Reactivation of Epstein-Barr Virus Lytic Cycle and Combinatorial Effects of Autophagy-Dependent and Independent Lytic Inducers in Nasopharyngeal Carcinoma
Source: Cancers (Basel). 2019 Nov 26;11(12):1871. doi: 10.3390/cancers11121871 (PMC6966612; doi:10.3390/cancers11121871)
Supplement: Supplementary file 1 [file cancers-11-01871-s001.pdf]

cancers

MDPI

Supplementary Materials

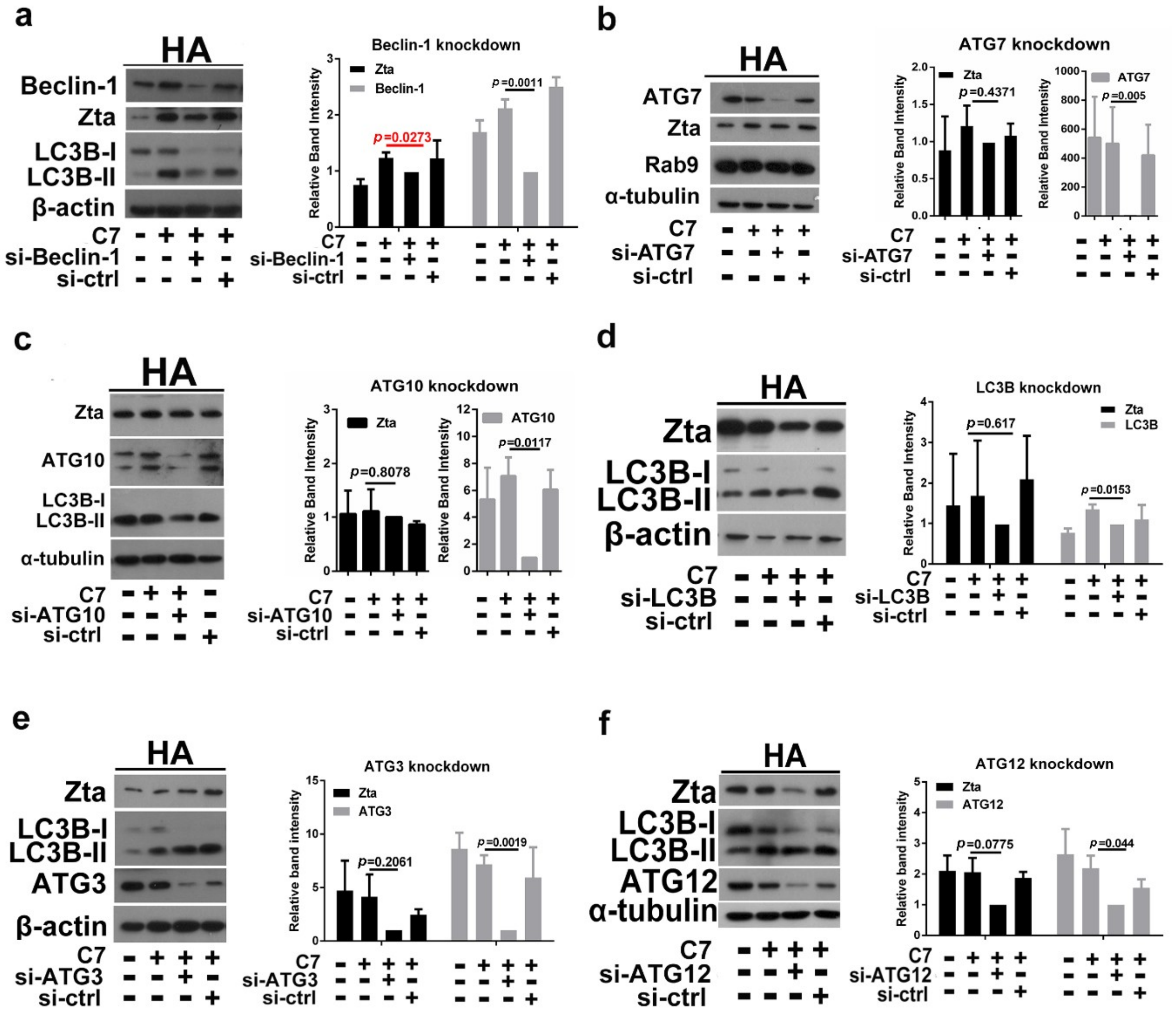

Figure S1. Autophagic protein ATG5 is specifically required for EBV lytic reactivation. (**a**) siRNA knockdown on Beclin-1 was performed in HA cells. Scramble siRNA was performed as a control. The cells were treated with 20 μM C7 for 2 h. The expression of Beclin-1, phosphorylated ERK1/2, Zta, Rab9 and LC3B was detected by Western Blot analysis. Cellular β-actin was detected as a loading control. (**b**) siRNA knockdown on ATG7 was performed in HA cells. Scramble siRNA was performed as a control. The cells were treated with 20 μM C7 for 2 h. The expression of ATG7, Zta and LC3B was detected by Western Blot analysis. Cellular α-tubulin was detected as a loading control. (**c**) siRNA knockdown on ATG10 was performed in HA cells. Scramble siRNA was performed as a control. The cells were treated with 20 μM C7 for 2 h. The expression of Zta, ATG10 and LC3B was detected by Western Blot analysis. Cellular α-tubulin was detected as a loading control. (**d**) siRNA knockdown on LC3B was performed in HA cells. Scramble siRNA was performed as a control. The cells were treated with 20 μM C7 for 2 h. The expression of ATG7, Zta and LC3B was detected by Western Blot analysis. Cellular α-tubulin was detected as a loading control. (**e**) siRNA knockdown on ATG3 was performed in HA cells. Scramble siRNA was performed as a control. The cells were treated with 20 μM C7 for 2 h. The expression of ATG3, Zta and LC3B was detected by Western Blot analysis. Cellular β-actin was detected as a loading control. (**f**) siRNA knockdown on ATG12 was performed in HA cells. Scramble siRNA was performed as a control. The cells were treated with 20 μM C7 for 2 h. The expression of ATG12, Zta and LC3B was detected by Western Blot analysis. Cellular α-tubulin was detected as a loading control. All of the above experiments were repeated and bands representing Zta and the knockdown protein were quantified and plotted as relative band intensity. Statistics were calculated with Student's T test.

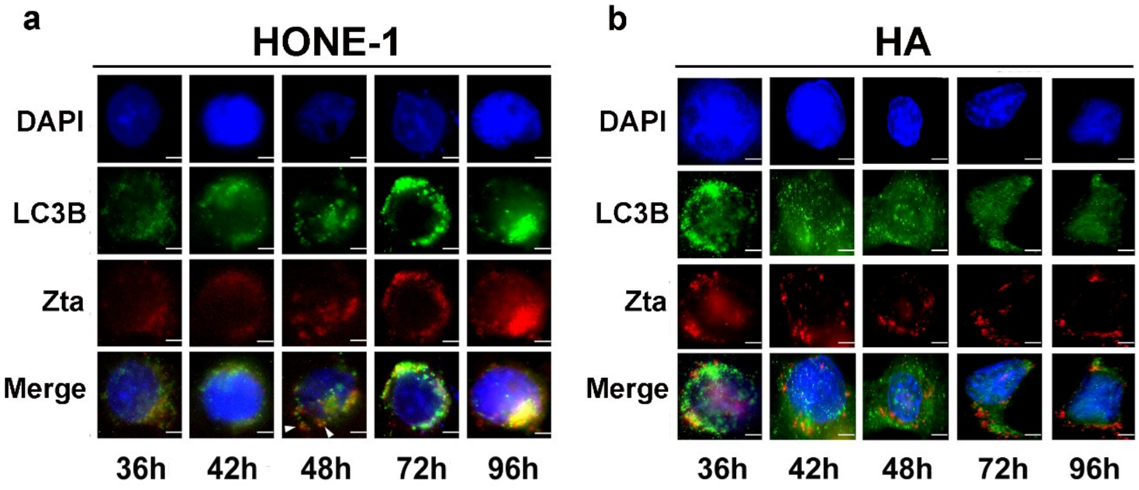

Figure S2. Positive feedback loop between Zta and autophagy initiation. (**a**) HONE-1 and (**b**) HA cells were treated with 20 μM C7 for 36, 42, 48, 72 or 96 h. Expression of lysosomal marker LAMP1 (red signals) and autophagosomes marker LC3B (green signals) was analyzed by immunofluorescence staining. DAPI (blue signals) stained cell nuclei. Scale Bar: 10 μm.

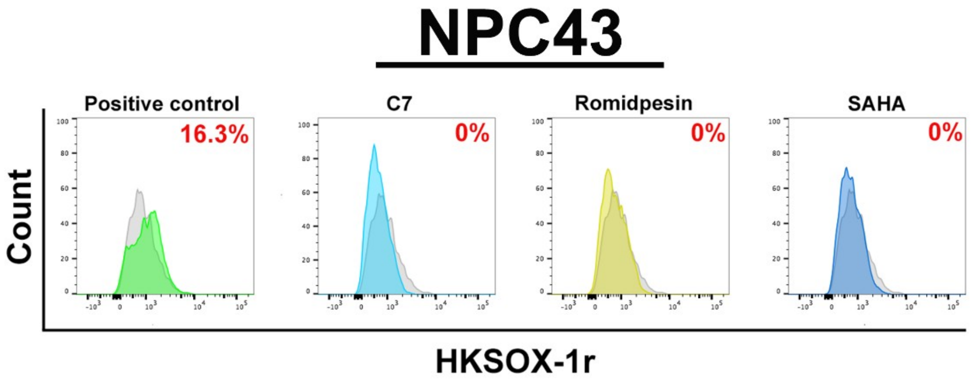

Figure 3. Cytosol superoxide is not related to EBV lytic reactivation upon C7 treatment. NPC43 cells were pre-incubated with 2 μM HKSOX-1r cytosol superoxide probe for 30 min. The cells were then either untreated or treated with 40 μM C7, 5nM romidepsin or 10 μM SAHA for 2 h, 10 μM Antimycin A was treated as a positive control. FITC signals were measured by flow cytometry and data were analyzed with FlowJo software.

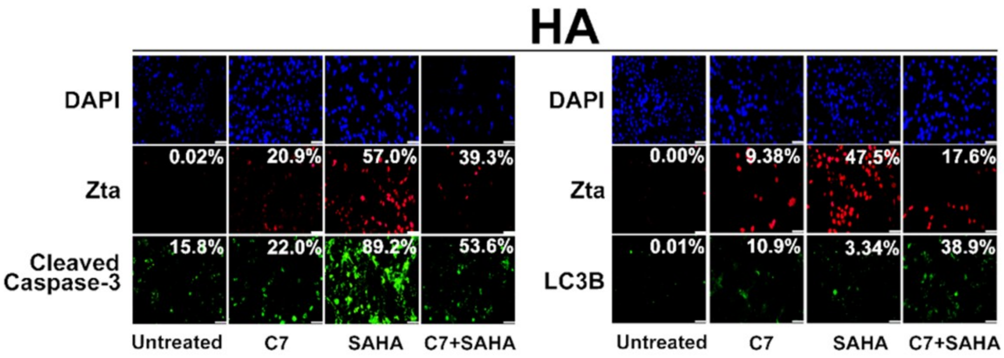

Figure S4. Enhancement of autophagy expression in HA cells resulted from the combinatorial effects of C7 and SAHA. HA cells were either untreated or treated with 20 μM C7, 10 μM SAHA or the combination of both compounds for 48 h. Expression of Zta (red signals), cleaved caspase-3 (left panel. green signals) and LC3B (right panel, green signals) was analyzed by immunofluorescence staining. DAPI (blue signals) stained cell nuclei. Scale Bar: 100 μm.

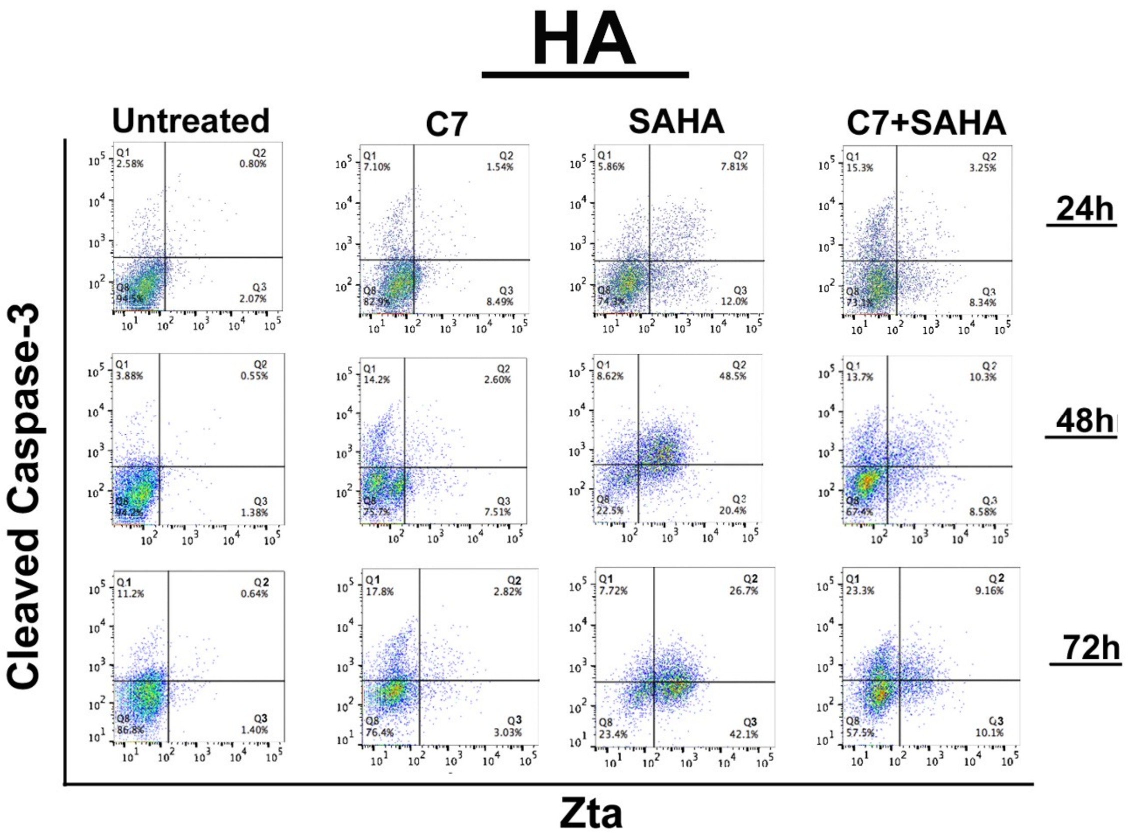

Figure S5. Quantification of cleaved caspase-3 and Zta expression by flow cytometry. HA cells were either untreated or treated with 20 μM C7, 10 μM SAHA or the combination of the two compounds for 24, 48, or 72 h. Percentage of cell death and the expression of CC3 and Zta was analyzed by flow cytometry as stated and data were analyzed with FlowJo software.

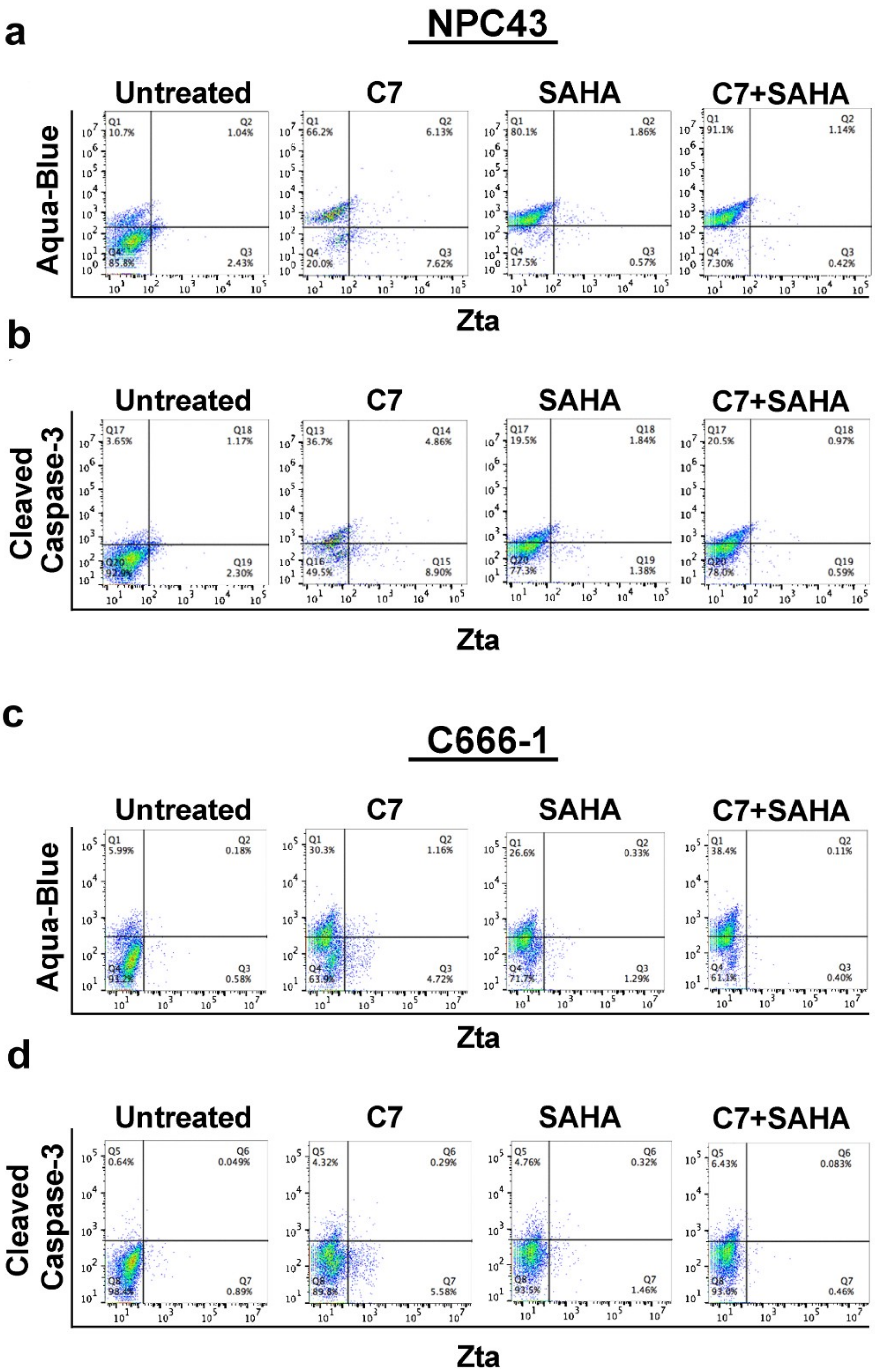

Figure S6. Quantification of cell death and expression of cleaved caspase-3 and Zta by flow cytometry. (**a**,**b**) NPC43 and (**c**,**d**) C666-1 cells were either untreated or treated with 40 μM C7, 20 μM SAHA or the combination of the two compounds for 72 h. Percentage of cell death and the expression of CC3 and Zta was analyzed by flow cytometry as stated and data were analyzed with FlowJo software.

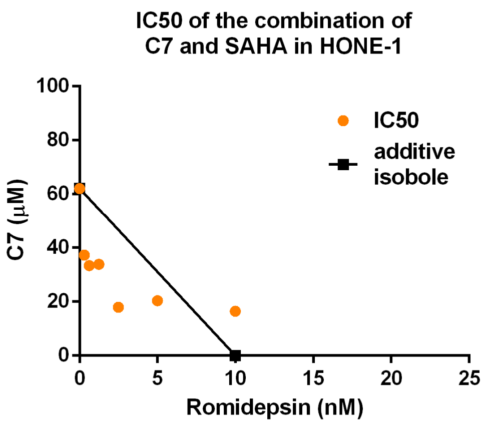

Figure 7. Non-specific killing in both EBV+ and EBV- NPC cells. HONE-1 cells were treated with a gradient combination of C7 and romidepsin for 48 h. Synergisms of proliferation inhibition of the two drugs in these cells were analyzed by isobologram analysis**.**

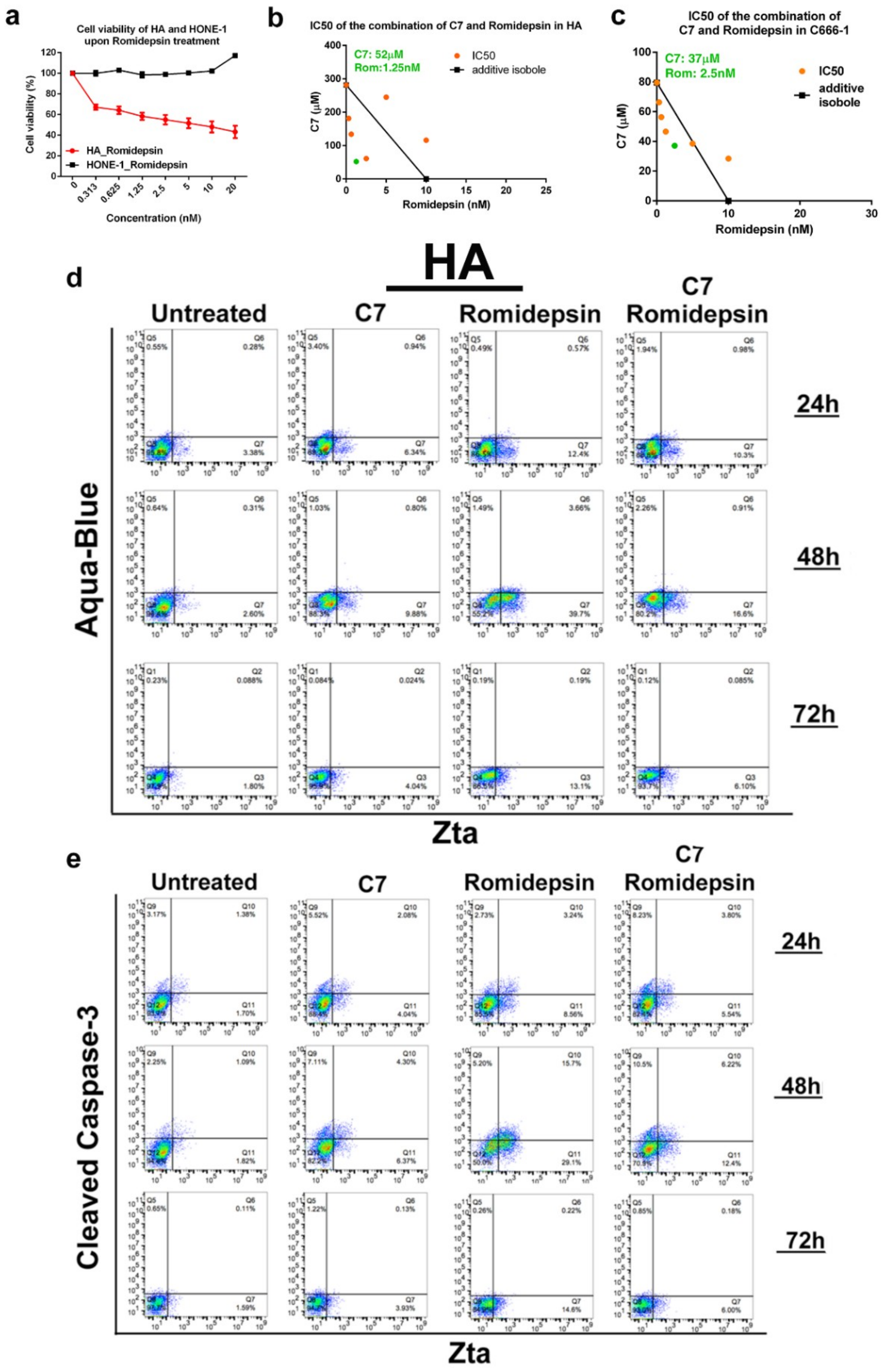

Figure S8. Antagonistic effect resulted from C7-romidepsin combination. (**a**) HA and HONE-1 cells were treated with 0, 0.313, 0.625, 1.25, 2.5, 5, 10 or 20 nM romidepsin for 48 h, were subjected to MTT assay and cell viability was plotted. (**b**) HA and (**c**) C666-1 cells were treated with a gradient combination of C7 and romidepsin for 48 hrs. Synergisms of proliferation inhibition of the two drugs in these cells were analyzed by isobologram analysis. (**d**,**e**) HA cells were either untreated or treated with 20 μM C7, 10 nM romidepsin or the combination of the two compounds for 48 h. Percentage of cell death and the expression of CC3 and Zta was analyzed by flow cytometry as stated and data were analyzed with FlowJo software.

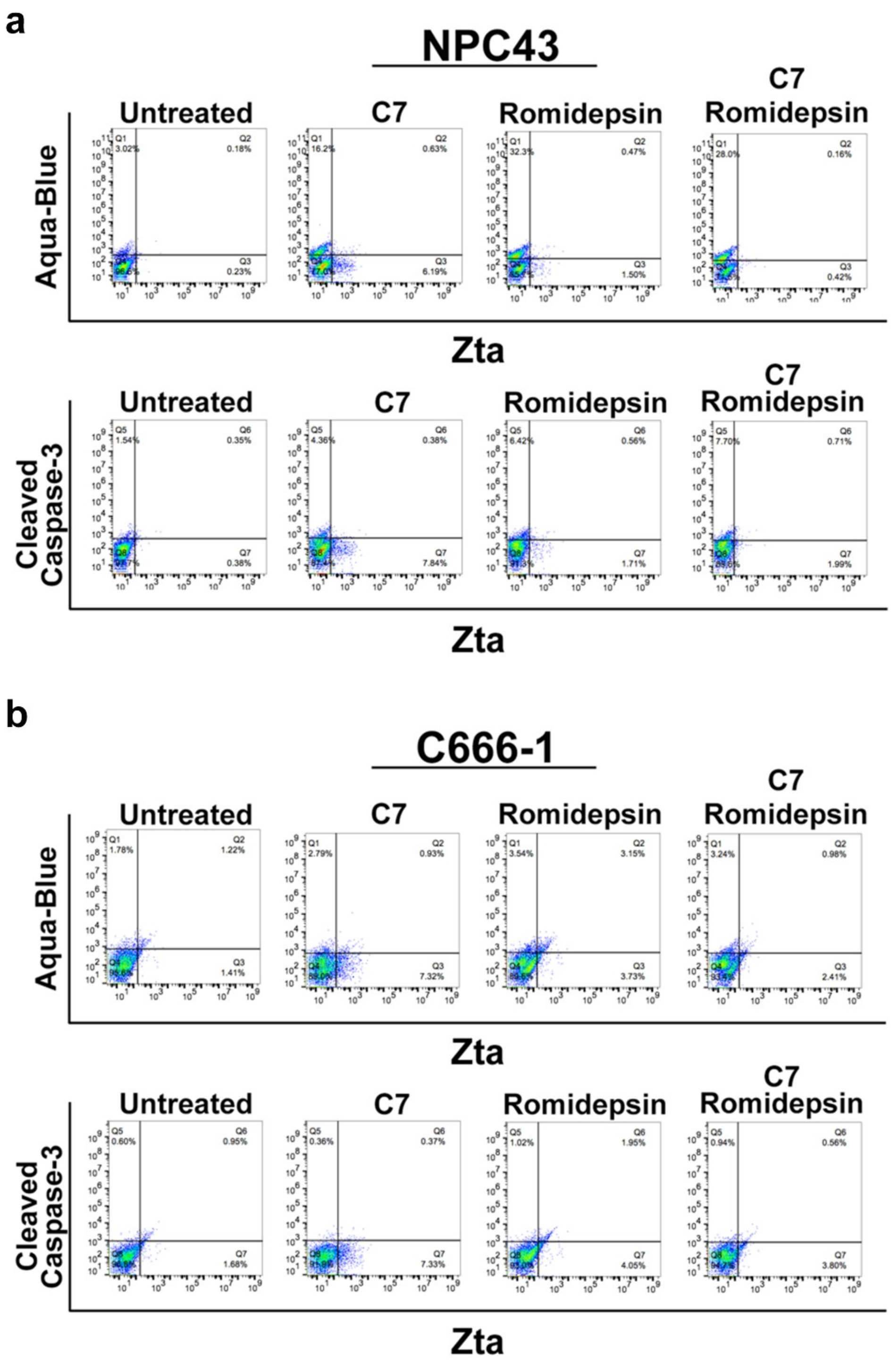

Figure S9. Quantification of cell death and expression of cleaved caspase-3 and Zta by flow cytometry. (**a**) NPC43 and (**b**) C666-1 cells were either untreated or treated with 40 μM C7, 10 nM romidepsin or the combination of the two compounds for 72 h. Percentage of cell death and the expression of CC3 and Zta was analyzed by flow cytometry as stated and data were analyzed with FlowJo software.

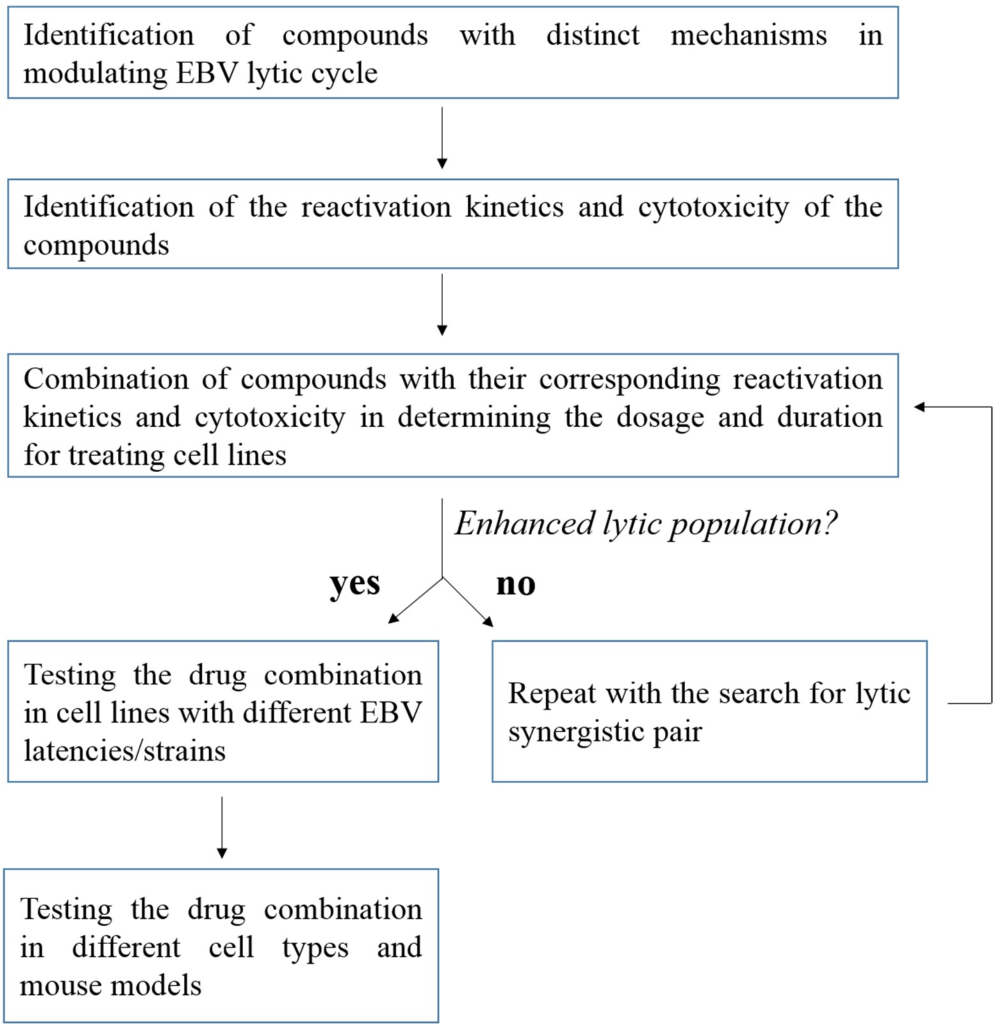

Figure S10. Strategic scheme for rational design of drug combination for EBV lytic reactivation.
